# Relationship between Muscle Mass/Strength and Hepatic Fat Content in Post-Menopausal Women

**DOI:** 10.3390/medicina55100629

**Published:** 2019-09-24

**Authors:** Yajie Zhang, Dajiang Lu, Renwei Wang, Weijie Fu, Shengnian Zhang

**Affiliations:** 1 School of Kinesiology, Shanghai University of Sport, Shanghai 200438, China; 2 Department of Human Movement Sciences, Vrije Universiteit Amsterdam, Amsterdam Movement Sciences, 1081HV Amsterdam, The Netherlands

**Keywords:** grip strength, knee extensors, sarcopenia, middle-aged women

## Abstract

*Background and Objectives*: Recent studies have shown that low skeletal muscle mass can contribute to non-alcoholic fatty liver disease through insulin resistance. However, the association between muscle mass/strength and hepatic fat content remains unclear in postmenopausal women. *Methods*: In this study, we assessed the associations between muscle mass/strength and various severities of non-alcoholic fatty liver disease. Using single-voxel proton magnetic resonance spectroscopy, 96 postmenopausal women between the ages of 50 and 65 were divided into four groups (G0–G3) by hepatic fat content: G0 (hepatic fat content <5%, *n* = 20), G1 (5% ≤ hepatic fat content < 10%, *n* = 27), G2 (10% ≤ hepatic fat content < 25%, *n* = 31), and G3 (hepatic fat content ≥25%, *n* = 18). Muscle mass indexes were estimated as skeletal muscle index (SMI)% (total lean mass/weight × 100) and appendicular skeletal muscular mass index (ASM)% (appendicular lean mass/weight × 100) by dual energy X-ray absorptiometry. Maximal isometric voluntary contraction of the handgrip, elbow flexors, and knee extensors was measured using an adjustable dynamometer chair. Fasting plasma glucose, insulin, and follicle-stimulating hormones were assessed in venous blood samples. *Results*: The results showed negative correlations between hepatic fat content and SMI% (r = −0.42, *p* < 0.001), ASM% (r = −0.29, *p* = 0.005), maximal voluntary force of grip (r = −0.22, *p* = 0.037), and knee extensors (r = −0.22, *p* = 0.032). *Conclusions*: These significant correlations almost remained unchanged even after controlling for insulin resistance. In conclusion, negative correlations exist between muscle mass/strength and the progressed severity of non-alcoholic fatty liver disease among post-menopausal women, and the correlations are independent of insulin resistance.

## 1. Introduction

The prevalence of non-alcoholic fatty liver disease (NAFLD) in China increased from 18.22% (2000–2006) to 20.86% (2010–2013) [1]. In recent years, the role of skeletal muscle in NAFLD has attracted increasing attention. On the one hand, skeletal muscle is the main site of insulin consumption, and muscular function impairment greatly contributes to the development of insulin resistance (IR) [2], which causes fatty liver disease [3]. As the muscle’s capacity to absorb glucose and synthesize glycogen decreases, carbohydrate-derived energy may divert into increased hepatic de novo lipogenesis [4,5], leading to fat accumulation in the liver. On the other hand, as a hallmark of NAFLD, IR may downregulate the effects of insulin on maintaining muscle mass. Insulin inhibits muscle protein degradation [6] and, together with amino acids, stimulates muscle protein synthesis [7,8]. Therefore, we hypothesized that among NAFLD patients with IR, imbalanced muscle protein synthesis and proteolysis could result in the loss of muscle mass, resulting in a vicious cycle.

The role of skeletal muscle mass in NAFLD has been addressed by a few studies. Moon et al. showed that high skeletal muscle mass may protect ectopic fat accumulation in the liver against NAFLD [9]. In addition, Hong et al. [10] and Kitajima et al. [11] reported that low muscle mass increases the risk of NAFLD. These discoveries suggest that skeletal muscle mass is associated with the development and progression of NAFLD.

Apart from muscle mass, muscle strength may also play a beneficial role among NAFLD patients. A limited number of interventional studies have observed enhanced knee extension strength in NAFLD patients after resistance exercise [12,13] or hybrid training of voluntary and electrical muscle contractions [14]. However, the association between muscle strength and NAFLD has not been completely investigated in these studies. Only one cross-sectional research compared muscle strength (quadriceps peak torque) in patients with a histological spectrum of NAFLD severities [15]. In consideration that skeletal muscle engenders physical forces as locomotion, which participates in the whole body’s energy metabolism, energy production and/or expenditure in skeletal muscle is likely to play an important role in maintaining energy homeostasis. Excessive energy would possibly be stored in the liver in the format of lipids, which increase hepatic fat accumulation [16].

Both muscle mass and muscle strength are potentially associated with the progression of NAFLD. However, such a relationship in certain groups of people with a certain age range and gender has not been clarified yet. The prevalence of NAFLD in females is higher than their counterparts across different age groups [17,18,19]. Furthermore, estrogen may have positive effects against NAFLD in women [20]. In previous studies concerning the role of skeletal muscle [9,10,11], male and female adults were combined without age subgroups. Therefore, their outcome of muscle mass could be limited to a specific age range and gender.

Therefore, this study investigated the relationship between hepatic fat content (HFC) and muscle mass/strength among middle-aged post-menopausal women with NAFLD and assessed whether various severities of NAFLD differ in muscle mass and muscle strength. The results of this study improve our understanding of the skeletal muscle’s importance in middle-aged postmenopausal women with NAFLD.

## 2. Methods

This cross-sectional study recruited 96 participants aged 50–65 years from the outpatient registration pool of the Yangpu District Health Care Service Center, in Shanghai, China. They were invited by doctors to fill in a questionnaire including alcohol consumption, health condition, and medication background and to check HFC by single-voxel proton magnetic resonance spectroscopy (^1^H MRS) [21]. The menopause state was defined by serum follicle-stimulating hormone (FSH) greater than 30 IU/L and last menstruation more than 6 months ago but within 10 years. Participants with body mass index (BMI) >38 kg/m^2^, cardiovascular issues, serious musculoskeletal problems, or mental illness were excluded. On the basis of HFC, the participants were classified into four groups: G0 (HFC < 5%, *n* = 20), G1 (5 ≤ HFC < 10%, *n* = 27), G2 (10 ≤ HFC < 25%, *n* = 31), and G3 (HFC ≥25%, *n* = 18).

This study was approved by the Ethics Committee of Association of Shanghai Nutrition (2013-003). All procedures and potential hazards were clarified to the participants in nontechnical terms, and informed consent was signed prior to the assessments.

HFC was quantified using ^1^H MRS (GE Sigma Excite HD CVI1.5T), localized below the second lumbar (L2) in supine position. HFC was analyzed using the linear combination of model spectra software, which is the standard for in vivo spectroscopy analysis [22,23].

Venous blood samples were obtained in standardized fasting conditions at 7:00–8:00 a.m. Fasting plasma glucose (FPG), insulin, and FSH were assessed using an automatic biochemistry analyzer and chemiluminescent immunoassay. IR was estimated using a homeostatic model assessment of insulin resistance (HOMA-IR) [24].

Dual energy x-ray absorptiometry (DXA Prodigy, GE Lunar Corp., Madison, WI, USA, software version: 13.60.033) was used to assess body composition. Lean mass was obtained from the whole-body scan. Skeletal muscle index (SMI)% was calculated as total lean mass/weight × 100, and appendicular skeletal muscular mass index (ASM)% was calculated as (arms lean mass + legs lean mass)/weight × 100.

The maximal isometric voluntary contraction of the right handgrip, left elbow flexors, and left knee extensors was measured using an adjustable dynamometer chair (Good Strength, IGS01, MetiturOy, Jyvaskyla, Finland) in a sitting position with the hips at a right angle. Grip strength was measured with a dynamometer fixed to the arm of the chair. Elbow flexion strength was measured with the elbow supported comfortably at 90°, and the thumb was placed in an upward position. The wrist was fastened by Velcro straps. Knee extension strength was measured at 120° with the ankle, thigh, and trunk fastened with Velcro straps. For each test, three trials were taken after one shot of about 50% level of maximal strength. During a trial, the participants were verbally encouraged to exert their maximal strength for 3 s. The three trails were separated by 30 s of rest time, and the maximal forces were recorded.

Data were checked for normality by Shapiro–Wilk’s test before each analysis using PASW statistics version 21 (IBM Corporation, Armonk, NY, USA). If data were not normally distributed, their natural logarithms were used. Descriptive data are shown as mean ± standard deviation (SD). Differences of variables among the four groups were tested using ANOVA and ANCOVA. A Pearson correlation and partial correlation were used for analyzing relationships. In all hypothesis tests, two-sided *p*-values of less than 0.05 were considered significant.

## 3. Results

The physical characteristics of the subjects are presented in Table 1. Body weight was higher in the participants with increased severities of NAFLD (G1 to G3) compared with the non-NAFLD (G0, all *p* < 0.001). G2 and G3 had higher BMI than G0 (*p* < 0.001 and *p* = 0.001), and G2 than G1 (*p* = 0.030). In addition, G2 and G3 had higher concentrations of insulin than G0 (*p* = 0.001 and *p* < 0.001) and G1 (*p* = 0.023 and *p* = 0.003, respectively). G2 and G3 also had higher levels of HOMA-IR than G0 (*p* = 0.007 and *p* = 0.001), and G3 than G1 (*p* = 0.014).

The strength of the handgrip, elbow flexion, and knee extension is shown in Table 2. G3 had lower hand grip force (Fgrip)/weight (Wt) than G0 (*p* = 0.023) and G1 (*p* = 0.008), and G2 had lower knee extension force (Fknee)/Wt than G1 (*p* = 0.039). G2 and G3 also had lower SMI% than G0 (*p* = 0.002 and *p* = 0.014), and G2 than G1 (*p* = 0.015). However, no significant differences were observed in ASM% among the four groups. After controlling for the HOMA-IR, the significant differences between the groups remained (*p* = 0.005–0.035). Interestingly, significant differences in Fknee/Wt and SMI% were observed between G3 and G1 (*p* = 0.016 and *p* = 0.030).

**Figure 1:**
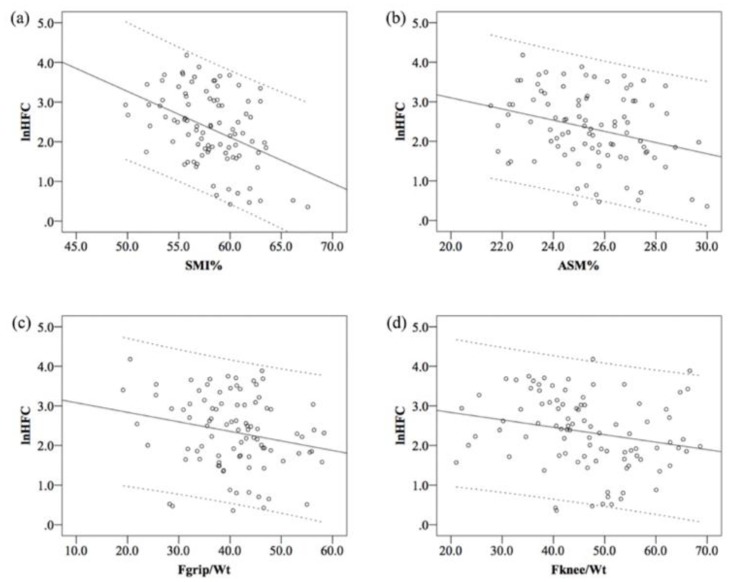
Relationship between lnHFC and SMI%, ASM%, Fgrip/Wt, and Fknee/Wt. Dotted lines represent 95% confidence interval.

HFC was negatively correlated with SMI% (r = −0.42, *p* < 0.001), ASM% (r = −0.29, *p* = 0.005), Fgrip/Wt (r = −0.22, *p* = 0.037), and Fknee/Wt (r = −0.22, *p* = 0.032) (Figure 1). Furthermore, HFC was positively correlated with the HOMA-IR (r = 0.39, *p* < 0.001). After controlling for the HOMA-IR, the significant relationships of HFC with muscle mass (r = −0.28 and −0.42, *p* < 0.001 and *p* = 0.009, respectively) and Fknee/Wt (r = −0.24, *p* = 0.022) remained, and the correlation between HFC and Fgrip/Wt (r = −0.20, *p* = 0.061) reached our criterion of significance. Moreover, FPG had no correlation with SMI% (r = 0.187, *p* = 0.070), whereas insulin was negatively correlated with SMI% (r = −0.25, *p* = 0.016).

## 4. Discussion

In this study, we explored the muscle mass and maximal isometric voluntary strength in middle-aged post-menopausal women with NAFLD. Moderate and severe NAFLD patients had low muscle mass and weak maximal isometric voluntary strength of handgrip and knee extensors. Moreover, the low quality of muscle was negatively correlated with the severity of NAFLD, and it was independent of IR.

Skeletal muscle and NAFLD may associate with each other via IR. In general, IR develops in target tissues (particularly muscle and liver) initially, followed by decreased insulin secretion. Insulin contributes to regulating hyperglycemia and inhibiting greater HFC by decreasing hepatic de novo lipogenesis [5]. It also contributes to regulating mitochondrial oxidative phosphorylation in muscles [25], which may result in decreased substrate oxidation and lipid accumulation [26], leading to metabolic diseases as NAFLD. The anabolic actions of insulin include stimulating muscle protein synthesis [7,8] and inhibiting muscle protein degradation [6]. Thus, a decreased anabolic action of insulin (caused by IR) may affect skeletal muscle. Moon et al. observed a moderate negative correlation between the fatty liver index and SMI% after adjusting for age [9]. In the present study, we observed similar results of the muscle mass being lower in moderate and severe NAFLD patients with greater IR, and HFC was negatively correlated with SMI%. Insulin was also negatively correlated with SMI%.

To rule out the influence of IR on the relationship between NAFLD and muscle mass, we controlled HOMA-IR and observed that the significant relationship remained. In other words, muscle status is associated with the development of NAFLD [27,28], independent of IR. Hong et al. also demonstrated that a high risk of NAFLD exists in individuals with low muscle mass after adjusting for IR and inflammation [10]. One explanation could be that the high muscle proportion of the body composition leads to high basal metabolic rate and energy expenditure. Another explanation could be the beneficial insulin sensitivity of muscle mass, which would compensate the lack of insulin to some degree. Studies have shown that high muscle mass is related to improved insulin sensitivity [29], and muscle dysfunction could impact insulin sensitivity and glucose metabolism [30]. However, we did not compare the insulin sensitivity among groups. The cross-sectional results of group comparisons and correlations suggested that high muscle proportion of the body composition could prevent NAFLD. However, how the muscle works on visceral fat accumulation remains to be fully understood.

Muscle strength is closely related to muscle function in daily life. Grip strength is a simple, inexpensive risk-stratifying method for all-cause death [31], and knee extension strength relative to body weight is well associated with self-reported difficulties and functional impairments [32]. When correlated with functional performance, muscle strength indexes (upper and lower relative muscle strength) are much stronger than ASM% [32].

We found that HFC was negatively correlated with Fgrip/Wt and Fknee/Wt, although these muscle strength indexes were not stronger than SMI% or ASM%. Our study showed that the knee extension strength was lower in the moderate and severe NAFLD groups than in the mild NAFLD group after controlling for IR. This result was consistent with the results that the negative correlation between HFC and Fknee/Wt existed independently of IR. However, another study showed no differences in isokinetic knee extension strength among various groups [15]. This finding might be attributed to the fact that the authors used the NAFLD activity score instead of HFC. When the same subjects were divided by severity of steatosis (mild, moderate, severe), a significant difference was found. In terms of handgrip strength, after controlling for IR, its negative correlation with HFC was not significant but the differences between the severe and mild NAFLD groups remained. Therefore, muscle strength decreased with higher HFC in post-menopausal women.

There are two possible reasons. First, the muscle is a secretory organ whose inactivity can cause an altered myokine response [33]. This altered response may have an association with NAFLD. Second, muscle activity (exerting force) consumes free fatty acids (FFA) in plasma, which is beneficial for preventing FFA uptake by hepatocytes [34]. Two longitudinal studies of hybrid exercise training (HYB) in NAFLD patients found elevated muscle strength after training. One emphasized the enlargement of intramyocellular space [35], the other emphasized the effects of HYB on intramyocellular lipid reduction [14]. Physical training may enhance muscle strength, associated with improved NAFLD conditions (e.g., lipid profile, decreased IR, and liver steatosis grade) [14]. Although those authors did not focus on the relationship between muscle strength and NAFLD characteristics, their results suggest that exploring the potential pathway and biomarkers is promising to link muscle quality and NAFLD in the future.

Our study has several limitations. First, not all of the maximal isometric muscle strength was measured from the dominated side. Considering that left-handed/footed participants were fewer than 0.1% of the whole, we assumed that the trend of muscle quality would not be affected. For most of the right-handed/footed participants, we measured the elbow flexors and knee extensors of the left side. Second, we did not consider the physical activity status of the participants. In addition, the current results do not apply to patients with non-alcoholic steatohepatitis (NASH). Meanwhile, it is unclear if similar results could be reproduced in other populations (for example in Western countries) due to the different genetic background of NAFLD/NASH [36]. Future research could focus on the possible biomarkers to further indicate the correlation and pathway between skeletal muscle and NAFLD progress.

## 5. Conclusions

Post-menopausal women with moderate and severe NAFLD tend to have low muscle mass, handgrip, and knee extensor strength. The results remained even after controlling for IR. Our study highlights the importance of improving muscle mass/strength in NAFLD patients.

## Figures and Tables

**Table 1:** Comparison of clinical and biochemical characteristics among groups with non-alcoholic fatty liver disease (NAFLD) by severity and without NAFLD.

	G0	G1	G2	G3
	(HFC < 5%)	(5 ≤ HFC < 10%)	(10 ≤ HFC < 25%)	(HFC ≥ 25%)
*N*	20	27	31	18
Age (years)	60.1 ± 3.9	60.2 ± 3.9	59.6 ± 3.7	58.7 ± 2.9
Height (cm)	159.1 ± 5.5	158.8 ± 5.2	158.1 ± 5.6	158.2 ± 8.1
Weight (kg)	60.9 ± 8.4	64.5 ± 8.4	68.1 ± 8.1 ^a^	68.0 ± 9.6 ^c^
BMI (kg/m^2^)	24.1 ± 2.8	25.6 ± 3.2	27.2 ± 2.5 ^a,b^	27.1 ± 2.7 ^c^
FPG (mmol/L)	5.61 ± 0.56	5.61 ± 0.69	5.43 ± 0.72	5.47 ± 0.77
Insulin (mIU/L)	11.27 ± 4.78	13.48 ± 7.66	18.18 ± 8.99 ^a,b^	21.47 ± 11.74^c,d^
HOMA-IR	2.83 ± 1.26	3.52 ± 2.17	4.55 ± 2.51 ^a^	5.05 ± 2.35 ^c,d^
FSH (mIU/mL)	65.3 ± 24.5	52.6 ± 15.5	51.9 ± 25.6 ^a^	44.8 ± 18.0 ^c^

^a^ Significant between G2 and G0, *p* < 0.05; ^b^ significant between G2 and G1, *p* < 0.05; ^c^ significant between G3 and G0, *p* < 0.05; ^d^ significant between G3 and G1, *p* < 0.05. Abbreviations: HFC, hepatatic fat content; BMI, body mass index; FPG, fasting plasma glucose; HOMA-IR, homeostatic model assessment of insulin resistance; FSH, follicle-stimulating hormone.

**Table 2:** Comparison of muscle mass and muscle strength by different HFC groups.

	G0	G1	G2	G3
	(HFC < 5%)	(5 ≤ HFC < 10%)	(10 ≤ HFC < 25%)	(HFC ≥ 25%)
N	20	27	31	18
Fgrip /Wt (%)	42.3 ± 7.2	42.8 ± 8.2	41.0 ± 8.0	36.3 ± 8.6 ^c,d^
Felbow/Wt (%)	29.0 ± 3.9	29.9 ± 4.6	27.7 ± 4.7	28.2 ± 5.1
Fknee /Wt (%)	48.6 ± 9.7	49.6 ± 12.1	43.7 ± 8.9 ^b^	43.4 ± 12.3
SMI (%)	59.6 ± 3.6	58.8 ± 2.8	56.7 ± 3.5^a,b^	57.0 ± 2.8 ^c^
ASM (%)	26.0 ± 2.1	25.6 ± 1.9	24.9 ± 1.8	25.0 ± 1.8

^a^ Significant between G2 and G0, *p* < 0.05; ^b^ significant between G2 and G1, *p* < 0.05; ^c^ significant between G3 and G0, *p* < 0.05; ^d^ significant between G3 and G1, *p* < 0.05. Abbreviations: SMI, skeletal muscle index; ASM, appendicular skeletal muscular mass index; Wt, weight; Fgrip, hand grip force; Felbow, elbow flexion force; Fknee, knee extension force.

## Abbreviations

^1^H MRS	single-voxel proton magnetic resonance spectroscopy
ASM%	appendicular skeletal muscular mass index; ASM% = appendicular lean mass/weight × 100%
BMI	body mass index
Felbow	elbow flexion force
FFA	free fatty acids
Fgrip	hand grip force
Fknee	knee extension force
FPG	fasting plasma glucose
FSH	follicle-stimulating hormone
HFC	hepatic fat content
HOMA-IR	homeostatic model assessment of insulin resistance
HYB	hybrid exercise training
IR	insulin resistance
NAFLD	non-alcoholic fatty liver disease
NASH	non-alcoholic steatohepatitis
SMI%	skeletal muscle index; SMI% = total lean mass/weight × 100%
Wt	weight
